# Leisure-time physical activity in youth as a predictor of adult leisure physical activity among Japanese workers: a cross-sectional study

**DOI:** 10.1186/s12199-017-0648-1

**Published:** 2017-04-07

**Authors:** Hiroaki Itoh, Fumihiko Kitamura, Noriko Hagi, Tomoe Mashiko, Takehisa Matsukawa, Kazuhito Yokoyama

**Affiliations:** 1 Department of Epidemiology and Environmental Health, Juntendo University Faculty of Medicine, 2-1-1 Hongo, Bunkyo-ku, Tokyo, 113-8421 Japan; 2 Yokkaichi Nursing and Medical Care University, Yokkaichi, Japan; 3 Clinical Research Support Center, The University of Tokyo Hospital, Tokyo, Japan

**Keywords:** Physical activity, Exercise, Predictor, Determinant, Childhood, Adulthood

## Abstract

**Background:**

Workers in Japan are not sufficiently active; however, it remains unclear how their leisure-time physical activity habits may be developed. This cross-sectional study investigated the relationship of age- and intensity-specific leisure-time physical activity in youth to adulthood leisure-time physical activity habits among Japanese workers.

**Methods:**

In 2012, 968 workers (333 males and 635 females) from three companies and six hospitals in the Tokai region of Japan agreed to complete and submit a self-administered questionnaire. Intensity-specific leisure-time physical activity at ages 12 and 20 years was assessed retrospectively, and workers’ current participation in regular leisure-time physical activity was assessed as an outcome measure. Multivariable-adjusted odds ratios (ORs) and 95% confidence intervals (CIs) were calculated using logistic regression analysis.

**Results:**

Mean ages for males and females were 40 and 37 years, respectively. Strenuous leisure-time physical activity at age 12 years was significantly positively associated with adulthood participation in leisure-time physical activity among male workers [adjusted OR (95% CI) = 2.29 (1.02, 5.14)]. Additionally, both strenuous and moderate physical activity at age 20 years was significantly positively associated with participation in regular leisure-time physical activity in adulthood among males and females.

**Conclusions:**

Our results suggest that some leisure-time physical activity in youth may predict adult workers’ participation in regular leisure-time physical activity in Japan. Encouragement of leisure-time physical activity in youth could therefore be an effective measure to develop adult leisure-time physical activity habits among workers.

## Background

Physical activity is associated with reduced risk of many chronic diseases [1] including cardiovascular diseases [2, 3], type 2 diabetes [4], and several cancers [5, 6], in addition to all-cause mortality [7]. Despite such apparent benefits of physical activity, a large proportion of adults in developed countries such as Japan do not participate in regular leisure-time physical activity. The National Health and Nutrition Surveys in Japan for 1997, 2004, and 2009 revealed that 70–80% of the working generation (aged 20–59 years) were not sufficiently active (i.e., were exercising less than 30 min twice a week) [8]. Because workers in Japan may not have enough leisure time or be in the habit of participating in regular leisure-time physical activity, it remains unclear how their leisure-time physical activity habits could be developed.

In Western countries, a non-negligible number of studies suggest the possibility that physical activity in childhood and youth is associated with future physical activity [9]. Such a “carryover” may also be effective for building workers’ leisure-time physical activity habits in Japan. However, previous studies have not reported consistent results [9–14]; therefore, further investigation is needed. Moreover, epidemiological evidence that considers specific age and intensity of leisure-time physical activity in youth and the relationship of these variables to leisure-time physical activity habits in adults is lacking, particularly in Asian countries. The Japanese mentality is unique and differs in potentially important ways from that of Western societies. Workaholism, obedience, tolerance and selfless devotion to country are typical Japanese tendencies that may influence workers’ behavioral priorities and their responses to health promotion efforts. Because it is also unclear whether leisure-time physical activity in youth predicts leisure-time physical activity in adulthood among Japanese people, an epidemiological study is needed. In addition, the optimal intensity level of leisure-time physical activity at specific ages that is necessary to develop future leisure-time physical activity habits remains to be determined. For example, experience of strenuous leisure-time physical activity in youth may build an individual’s physical strength and enable them to resume leisure-time physical activity in adulthood.

The purpose of the present study was to investigate, in a sample of Japanese workers, the associations between age- and intensity-specific leisure-time physical activity in youth and participation as an adult in regular leisure-time physical activity. Whereas most previous studies have examined the association between total activity in youth and total activity in adulthood [10], this study focused on leisure-time physical activity. This will provide further insights for promoting a more desirable physical activity strategy, because leisure-time activity is modifiable and controllable. Furthermore, health effects of leisure-time physical activity may differ from those of work-related physical activity. In fact, higher occupational physical activity has recently been shown to have acute and chronic adverse effects, such as increased risk of cardiovascular disease and all-cause mortality, particularly among men [15]. Therefore, our independent evaluation of leisure-time physical activity will be informative. We used ages 12 and 20 years as representative time points for leisure-time physical activity in youth, based on the following precedents. In previous studies conducted in Australia [10] and Canada [16] that tracked physical activity, the mean age at baseline was 12 years. In another tracking study conducted in Belgium [17], the mean ages of the male and female follow-up samples at baseline was 20 and 22 years, respectively.

Leisure-time physical activity is defined as “physical activity performed during exercise, recreation, or any time other than that associated with one’s regular occupation, housework, or transportation” [18, 19], and as “primarily exercise or sports-related activities” [20]. “Exercise and other forms of physical training are types of recreational physical activity” [21]. Therefore, “leisure-time physical activity” may be synonymous with “exercise.” These two terms were treated impartially in the present study.

## Methods

### Participants

In 2012, we conducted a cross-sectional study in three companies (railway, transport, and driving school) and six hospitals in the Tokai region of Japan. We distributed a paper questionnaire to nearly all employees (mean 92%) at these workplaces. Of the 1,489 employees, 968 agreed to participate in the study (response rate 65%) and submitted completed questionnaires. The questionnaire’s instructions informed respondents that their participation was voluntary and that completing and returning the questionnaire would signify their agreement to participate. The sample comprised 333 males and 635 females (mean ± SD of age were 40.0 ± 10.6 and 37.0 ± 11.7 years, respectively). Many of the participating hospital workers were nurses and practical nurses. The study procedure was approved in advance by the Institutional Review Board of the Juntendo University Faculty of Medicine, Tokyo, Japan (receipt number 813; approval letter number 2012057; May 21, 2012).

### Data collection using questionnaires

The self-administered questionnaire collected information such as socio-demographic and anthropometric characteristics; lifestyle, including leisure-time physical activity; and medical history. Body mass index was calculated as weight in kilograms divided by the square of height in meters.

### Exposure assessment of youth leisure-time physical activity

Intensity-specific leisure-time physical activity at 12 and 20 years was retrospectively assessed by the questionnaire. Details of this questionnaire have previously been described elsewhere [22]. With regard to intensity, the questionnaire first asked whether they participated in “strenuous leisure-time physical activity,” with running, basketball, competitive swimming, and gymnastics provided as examples, as well as “moderate leisure-time physical activity” such as volleyball, softball, walking, and cycling. Participants were also asked to indicate how often they participated in the chosen activities, using the following six frequency categories: less than once per month, 1–3 days per month, 1–2 days per week, 3–4 days per week, 5–6 days per week, or every day of the week. For analyses, participation was transformed into a dichotomous variable: No (none) or Yes (which included “less than once per month,” “1–3 days per month,” “1–2 days per week,” “3–4 days per week,” “5–6 days per week,” and “every day of the week”). This was because the number of workers with current participation in leisure-time physical activity in the present study was limited. In addition, history of participation in sports club activities, including organized school sports during preschool age, elementary school, junior high school, high school or higher professional school, and junior/technical college or university was also assessed. Although sports club activities may partly overlap the above-mentioned leisure-time physical activities at ages 12 and 20 years, they were assessed separately.

### Outcome assessment of workers’ leisure-time physical activity habits

The sole outcome of the present study was leisure-time physical activity in adulthood. Here, “regular leisure-time physical activity” was defined as exercising at least twice a week for 30 min per session for more than 1 year. This definition was employed in the National Health and Nutrition Survey in Japan (Ministry of Health, Labour and Welfare of Japan).[8] Participants answered this single question with “yes” or “no.”

### Statistical analysis

All statistical analyses were performed with SAS software version 9.2 for Windows (SAS Institute Inc., Cary, NC, USA). One woman who did not provide her age, height, and weight, and reported an abnormally high physical activity level was excluded from the analysis. For comparisons of workers’ characteristics according to current participation in leisure-time physical activity, we used Wilcoxon rank sum test for continuous variables and Fisher’s exact probability test for categorical variables. Unconditional logistic regression analysis was performed to calculate multivariable-adjusted odds ratios (ORs) and 95% confidence intervals (CIs) using the SAS LOGISTIC procedure. In this analysis, workers’ current participation in leisure-time physical activity was entered as a dependent variable, while leisure-time physical activity in youth was entered as an independent variable. The ORs were adjusted for age (continuous variable) and education (dummy variable). We considered these variables to be potential confounders. Educational level was controlled because of a recent related finding [23]. However, we did not consider the number of holidays as a potential confounder based on causal diagrams [24]; indeed, the number of holidays availed by the workers in recent times would not affect their leisure-time physical activity habits in youth. All analyses were performed by sex, and observations with missing or disallowed values were not used for multivariable analyses (listwise case deletion). All p values and 95% CIs were two-sided, and significance was accepted at p < 0.05. When we used leisure-time physical activity at age 20 years as an exposure variable, the participants under 20 years of age (four men and one woman) were excluded from the analysis.

## Results

Table 1 shows participants’ basic characteristics according to current participation in leisure-time physical activity by sex. There were no significant differences by current participation in leisure-time physical activity, with the exception of the number of holidays. Women participating in current leisure-time physical activity had more holidays than women without current participation in leisure-time physical activity, and women had more holidays than men.

**Table 1:** Characteristics of study participants

	Males			Females		
	Current participation in regular leisure-time physical activity^a^			Current participation in regular leisure-time physical activity^a^		
Characteristics	Yes	No	p^b^	Yes	No	p^b^
N	33	271		42	534	
Age [years], mean ± SD	43.1 ± 11.1	39.8 ± 10.4	0.18	39.5 ± 12.0	36.5 ± 11.3	0.099
Body mass index [kg/m^2^], mean ± SD	23.2 ± 2.9	23.6 ± 4.0	0.78	23.6 ± 4.0	21.5 ± 3.7	0.75
Monthly overtime hours [hours], mean ± SD	27.7 ± 26.1	35.1 ± 27.3	0.16	6.4 ± 5.2	8.7 ± 11.2	0.80
Sleep duration [hours], mean ± SD	6.4 ± 1.0	6.3 ± 1.0	0.48	6.2 ± 1.7	6.4 ± 1.1	0.20
Number of holidays [days/month], mean ± SD	7.7 ± 1.8	7.1 ± 2.0	0.16	9.7 ± 3.0	9.0 ± 2.2	0.025
Length of service [years], mean ± SD	15.8 ± 11.4	15.7 ± 10.4	0.86	13.4 ± 9.9	12.5 ± 10.1	0.39
GHQ-12 score, mean ± SD	3.5 ± 3.2	3.8 ± 3.4	0.69	4.1 ± 3.6	4.0 ± 3.3	0.99
Educational level, n (%)			0.58			1.00
High school graduate	10 (32.3)	80 (31.6)		6 (15.4)	77 (14.8)	
Junior/technical college graduate	8 (25.8)	48 (19.0)		26 (66.7)	351 (67.4)	
University degree or higher	13 (41.9)	125 (49.4)		7 (18.0)	93 (17.9)	
Married, n (%)	22 (66.7)	186 (69.9)	0.69	22 (55.0)	263 (49.6)	0.62
Night-shift worker, n (%)	13 (40.6)	74 (28.4)	0.16	32 (78.1)	392 (74.8)	0.71
Current smoker, n (%)	7 (21.9)	106 (40.0)	0.054	8 (19.5)	56 (10.7)	0.12
Regular drinker, n (%)	14 (43.8)	125 (46.8)	0.85	11 (26.8)	111 (21.3)	0.43
Any unusual item in the results of the latest physical examination, n (%)	17 (54.8)	141 (56.4)	1.00	19 (45.2)	222 (45.5)	1.00
History of type 2 diabetes, n (%)	1 (3.3)	12 (5.3)	1.00	1 (2.8)	8 (1.8)	0.50
History of hypertension, n (%)	6 (19.4)	35 (15.1)	0.60	2 (5.6)	33 (7.3)	1.00
History of depression, n (%)	3 (10.0)	11 (4.9)	0.22	2 (5.6)	15 (3.3)	0.36

Number of subjects varied across variables as a result of missing information

*GHQ-12* General Health Questionnaire 12-item scale [28], *SD* standard deviation

^a^Defined as exercising at least twice a week for 30 min per session for more than 1 year

^b^Wilcoxon rank sum test used for continuous variables and Fisher’s exact probability test used for categorical variables

**Fig. 1:**
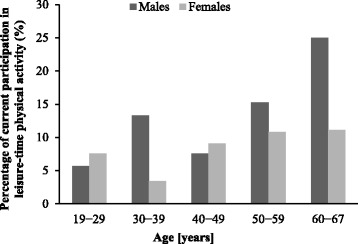
Prevalence of regular leisure-time physical activity among workers by age group. Regular leisure-time physical activity: defined as exercising at least twice a week for 30 min per session, for more than 1 year

Figure 1 shows the percentages of workers with current participation in regular leisure-time physical activity by age group. There was a higher proportion of participation in older age groups (50s and 60s).

Table 2 shows the ORs and 95% CIs for workers’ current participation in regular leisure-time physical activity by intensity levels of age-specific leisure-time physical activity. We found that strenuous leisure-time physical activity at age 12 years was significantly positively associated with current participation in leisure-time physical activity among male workers [adjusted OR (95% CI) = 2.29 (1.02, 5.14)]. In contrast, female leisure-time physical activity at age 12 years was not significantly associated with adulthood participation in regular leisure-time physical activity. Moreover, strenuous and moderate leisure-time physical activity at age 20 years were also significantly positively associated with workers’ current participation in regular leisure-time physical activity, and this held true for both males and females.

**Table 2:** Current participation in leisure-time physical activity according to intensity levels of age-specific leisure-time physical activity among Japanese workers

Leisure-time physical activity		Current participation in regular leisure-time physical activity^a^					
		Males			Females		
		Yes	No	Adjusted OR (95% CI)^b^	Yes	No	Adjusted OR (95% CI)^b^
At age 12 years							
Strenuous^c^	No	15	158	1.00 (reference)	26	357	1.00 (reference)
	Yes	18	109	**2.29 (1.02, 5.14)**	15	169	1.40 (0.69, 2.81)
Moderate^d^	No	12	119	1.00 (reference)	20	286	1.00 (reference)
	Yes	18	144	1.27 (0.56, 2.86)	22	225	1.50 (0.77, 2.92)
At age 20 years							
Strenuous^c^	No	20	203	1.00 (reference)	29	487	1.00 (reference)
	Yes	13	61	**2.35 (1.04, 5.27)**	11	42	**4.93 (2.18, 11.13)**
Moderate^d^	No	13	175	1.00 (reference)	26	415	1.00 (reference)
	Yes	17	86	**2.63 (1.16, 5.94)**	16	103	**2.37 (1.17, 4.79)**

Bold type indicates statistically significant values (p < 0.05)

*OR* odds ratio, *CI* confidence interval

^a^Defined as exercising at least twice a week for 30 min per session for more than 1 year

^b^Unconditional logistic model adjusted for age (continuous) and education (high school, junior/technical college, university or higher)

^c^Exemplars include running, playing basketball, swimming competitively, or doing gymnastics

^d^Exemplars include playing leisure-time volleyball, playing softball, walking, or cycling

Table 3 shows ORs and 95% CIs for workers’ current participation in regular leisure-time physical activity by history of participation in sports club activities at a young age. Female participation in such activities during high school or higher professional school was significantly positively associated with their current participation in regular leisure-time physical activity [adjusted OR (95% CI) = 3.21 (1.54, 6.68)]. In addition, female sports club activity in junior/technical college or university was also associated with participation in adulthood leisure-time physical activity. Point estimates for other ORs ranged from 1.16 to 2.18, although they were statistically insignificant.

**Table 3:** Current participation in leisure-time physical activity according to history of participation in sports club activities among Japanese workers

History of participation in sports club activities^a^	Current participation in regular leisure-time physical activity^b^					
	Males			Females		
	Yes	No	Adjusted OR (95% CI)^c^	Yes	No	Adjusted OR (95% CI)^c^
During preschool						
No	27	212	1.00 (reference)	33	437	1.00 (reference)
Yes	6	59	1.42 (0.50, 4.07)	9	92	1.70 (0.70, 4.19)
During elementary school						
No	16	124	1.00 (reference)	26	334	1.00 (reference)
Yes	17	147	1.17 (0.49, 2.81)	16	196	1.32 (0.64, 2.74)
During junior high school						
No	3	52	1.00 (reference)	8	127	1.00 (reference)
Yes	30	217	2.03 (0.58, 7.07)	34	405	1.24 (0.56, 2.78)
During high school or higher professional school						
No	9	120	1.00 (reference)	12	296	1.00 (reference)
Yes	22	146	2.18 (0.95, 5.03)	28	225	**3.21 (1.54, 6.68)**
During junior/technical college or university						
No	13	133	1.00 (reference)	24	393	1.00 (reference)
Yes	6	55	1.16 (0.41, 3.31)	11	67	**2.48 (1.10, 5.61)**

Bold type indicates statistically significant values (p < 0.05)

*OR* odds ratio, *CI* confidence interval

^a^Including both school clubs and sports clubs outside of school

^b^Defined as exercising at least twice a week for 30 min per session for more than 1 year

^c^Unconditional logistic model adjusted for age (continuous) and education (high school, junior/technical college, university or higher)

The direction and significance of the ORs in Tables 2 and 3 were unchanged after additionally adjusting for type of business (railway, transport, driving school, and hospital), night shift work, and the number of holidays (data not shown), with the exception of male sports club activity during high school or higher professional school [adjusted OR (95% CI) = 2.54 (1.04, 6.21)].

## Discussion

Among Japanese workers, some leisure-time physical activities in youth were significantly positively associated with participation in regular leisure-time physical activity in adulthood. In particular, male strenuous leisure-time physical activity at 12 years old was positively associated with adulthood leisure-time physical activity. Strenuous and moderate leisure-time physical activities at age 20 years were also predictive of workers’ current leisure-time physical activity behavior. Associations were evident and most were relatively strong in magnitude and consistent in direction. To our knowledge, this is the first study to investigate the association between leisure-time physical activity in youth and in adulthood among workers in an Asian country, also taking age- and intensity-specific activity into account. Although we only considered leisure-time physical activity, exposure in youth was a predictor of adult leisure-time physical activity in our Japanese sample. There is supportive evidence from a study in Australia reporting that childhood sport participation predicted adult physical activity, while total physical activity in childhood did not predict adult activity [10].

In this study, workers were distributed over a wide age range. Workers in older age groups tended to participate in regular leisure-time physical activity. This tendency is consistent with that observed in Japan’s National Health and Nutrition Survey [8]. Such change in behavior might be attributed in part to health consciousness and increased leisure time, given that those in older age groups no longer needed to care for young children.

Different intensities of leisure-time physical activity in youth were predictive of future leisure-time physical activity behavior. For males, strenuous leisure-time physical activity at age 12 years had a greater lingering effect than did moderate activity. Plausibly, children who engaged in strenuous leisure-time physical activity would gain physical strength more easily, enabling them to participate in future leisure-time physical activity. Additionally, because moderately intense leisure-time physical activity is often more accessible, safer, and more convenient, this level of activity at age 20 years may easily lead to lifelong participation. Our age- and intensity-specific analyses revealed that both strenuous and moderate intensity leisure-time physical activity at age 20 years was effective in developing future leisure-time physical activity habits. Thus, an age- and intensity-specific analysis can provide useful insights for leisure-time physical activity promotion strategies.

A sex difference in the results at age 12 was clearly observed. Such weaker associations in females may be attributed in part to major transitions in their life course, such as the transition from schooling to employment, from singlehood to marriage and having children, or experiencing unemployment [9]. Such events usually have different influences, particularly on continuous employment, for males and females in Japan. These findings regarding sex difference are supported by evidence from the majority of previous studies in Western countries. These studies also tend to show that childhood physical activity positively predicts adulthood physical activity in males, whereas about half of the associations are non-significant in females [9]. This tendency may be common in developed countries beyond the sphere of their residential area and local mindset.

Females’ sports club activity during high school or above was significantly positively associated with regular participation in adult leisure-time physical activity. This was not the case for males, although the point estimate of the OR was high. The small sample size of the subgroup might have led to the null result. Furthermore, with regard to sports club activities prior to high school in both male and female participants, no significant associations were seen. It is known that physical activity in the distant past generally shows a weaker correlation with adulthood physical activity than does activity the near past [11]. Another possible explanation for the lack of a significant association between sports club activities at a younger age and participation in leisure-time physical activity in adulthood is that some students who did not participate in organized school sports might have participated in other leisure-time physical activities and physical education. Although a previous study conducted in the United States [13] reported that the frequencies of forced and encouraged exercise during the preteen years were inversely associated with adult physical activity, such an association was not observed in the present study. Therefore, sports club activity may also be recommended for young people in Japan.

From a descriptive perspective, Tables 2 and 3 show that most workers who had participated in leisure-time physical activity in youth were not currently participating in regular leisure-time physical activity. For example, of the 127 male workers who had participated in strenuous leisure-time physical activity at age 12 years, 109 did not exercise regularly in adulthood. This suggests that continuing or resuming leisure-time physical activity in adulthood might not have been easy for the participating workers. Further studies are needed to investigate the kind of work environment that supports their participation in leisure-time physical activity.

Major strengths of the present study were as follows. Our questionnaire for assessing exposure to leisure-time physical activity at a young age took into consideration the intensity, frequency and timing of leisure-time physical activity. Workers’ current participation in regular leisure-time physical activity was clearly defined in terms of frequency and duration. Whereas most previous studies have examined the association between total activity in youth and in adulthood, the present study focused on leisure-time physical activity.

Several potential limitations of the study also warrant mention. First, we carried out multiple comparisons, which might have led to some false-positive results. However, we did not apply the Bonferroni correction because we considered this method too conservative. Second, owing to the cross-sectional nature of this investigation and self-reported measures of leisure-time physical activity habits in youth, we cannot completely rule out the possibility of error owing to recall bias. However, the reliability of long-term recall of participation in physical activity has been investigated in the United States and modest correlations have been reported [25]. Third, the response rate was suboptimal. However, the evidence for the relationship between low response rate and non-response bias is generally weak [26]; therefore, our moderate response rate may not necessarily indicate non-response bias. Fourth, the sample in the present study may not be representative of the general working population in Japan; therefore, our findings may not be applicable to a wide range of Japanese workers, although the observed associations were similar to those in previous studies. Although a disproportionate number of female participants (74.8%) were night-shift workers, this type of work is common in modern society. In developed countries, about 20% of the working population is engaged in shift work [27]. More detailed information regarding the type of work may provide further insights when assessing these associations. In addition, our sample size was not large enough to allow dose–response analysis using the frequency of leisure-time physical activity in youth, as well as subgroup analyses by age groups. A larger study sample that enables such analyses should be considered for future studies. In addition, timing of assessment of leisure-time physical activity in youth at ages other than 12 and 20 years may be useful, as the effects of leisure-time physical activity at other ages might differ from those in transitional years such as ages 12 and 20 years.

## Conclusions

In conclusion, our results suggest that certain aspects of leisure-time physical activity in youth may predict current participation in regular leisure-time physical activity in Japanese workers. Nurturing leisure-time physical activity in youth may be an effective measure to develop the future leisure-time physical activity habits of adult workers.

## Abbreviations

CI: Confidence interval
OR: Odds ratio
